# Ketamine‐induced uropathy: A narrative systemic review of surgical outcomes of reconstructive surgery

**DOI:** 10.1002/bco2.239

**Published:** 2023-04-19

**Authors:** Gabriel Vizgan, Michael Huamán, Kevin Rychik, Max Edeson, Jerry G. Blaivas

**Affiliations:** ^1^ SUNY Downstate Brooklyn New York USA; ^2^ Department of Urology Institute for Bladder and Prostate Research New York New York USA; ^3^ Duke University Medical Center Durham North Carolina USA; ^4^ Hackensack Meridian School of Medicine New Jersey USA; ^5^ Department of Urology Mount Sinai Health System New York New York USA

**Keywords:** drug abuse, end‐stage bladder disease, ketamine, ketamine cystitis, LUTS, meta‐analysis, reconstructive surgery

## Abstract

**Aims:**

Refractory ketamine‐induced uropathy (KU) (RKU) has devastating effects on the lower urinary tract leading to ureteral obstruction and even renal failure. The only effective treatment for RKU is major surgical reconstruction or urinary diversion. Nevertheless, there is a paucity of awareness about this destructive condition; the aim of this study is to conduct a narrative systemic review of all surgical outcomes of RKU.

**Methods:**

This is an English language literature review of surgical outcomes in KU patients who underwent reconstructive lower urinary tract surgery or urinary diversion through 5 August 2022. Two independent researchers assessed the relevance of each paper and disputes were settled by a third party. In‐vitro, animal studies, letters to the editor and papers that did not evaluate surgical outcomes were excluded.

**Results:**

Of the 50 763 identified articles, 622 were relevant based on title, 150 based on abstract, but only 23 papers were relevant by content. In all, 875 patients were documented as having KU, of whom 193 (22%) underwent reconstructive surgery. The data were disconcerting, as the apparent rapid progression from the beginning of KU to end‐stage bladder was only a 1‐year difference of ketamine abuse between those patients who required surgery (4.4 years) and those that did not (3.4 years).

**Conclusions:**

The data suggest that the time interval from the beginning of ketamine‐induced uropathy to the end‐stage bladder may be measured in months, confounding decision making. There is a dearth of literature about KU, and more research is needed to better understand this pathology.

## 1 INTRODUCTION

Discovered in 1960, ketamine hydrochloride is a glutamatergic N‐methyl‐D‐aspartate antagonist that has been used for decades as a short‐acting, but powerful, dissociative anaesthetic employed in both human and veterinary medicine.^1^ However, its use as an anaesthetic has been limited due to its hallucinogenic side effects, which have been described as a ‘near death’ or ‘out of body’ experience and generally occur during waking.^2^ Known as ‘K’, ‘Special K’ or ‘Dorothy’ on the street, ketamine has become a popular recreational drug because of the powerful hallucinations and out‐of‐body experiences it affords. Furthermore, due to the misconception that ketamine is less addictive or potentially harmful than other black‐market drugs, the use of street ketamine has increased over the years.^3^ This, coupled with its availability (retailing at £20 per gram compared with £50 per gram for cocaine and £45 per gram for heroin, on the black market),^4^ has led to cases of ketamine‐associated pathology becoming more prevalent. In 2006, ketamine was made a Class C substance via the Misuse of Drugs Act, and in 2014, 1.8% of individuals aged 16–24 years reported using ketamine in the last year.^3^ Recreational use of ketamine can cause very serious injury and dependence. Common side effects include memory loss, depression, thought dissociation, delusional thinking, abdominal cramps and cystitis‐like symptoms.^5^

Recently, there has been an emergence of data reporting a high prevalence of a symptom complex characterized by severe lower urinary tract symptoms (LUTS) including urinary frequency and urgency, small volume voids, painful haematuria and a biopsy revealing inflammation that resembled interstitial cystitis in those patients abusing ketamine.^6^, ^7^, ^8^ In one study, approximately two thirds of ketamine abusers had at least one LUTS, while only 19% of control subjects had these symptoms.^9^ This symptom complex has come to be termed ‘ketamine‐induced uropathy’ (KU),^10^ and its end stage is devastating usually requiring major surgical interventions.

However, given the destructive nature of end‐stage KU, there is still a dearth of information about this symptom complex in the literature. In order to raise awareness of the disease amongst the public and medical communities, this systemic review and meta‐analysis sought to compile all of the data available in the literature regarding outcomes of reconstructive surgery in those suffering from end‐stage KU.

## 2 METHODS

This is an English language literature review of surgical outcomes in KU patients who underwent reconstructive lower urinary tract surgery (RLUTS) or urinary diversion through 5 August 2022. Two independent researchers read each paper's title and if both found the paper to be relevant, its abstract was read and then, after applying exclusion criteria, the full papers were read; all disagreements regarding relevance were settled by a vote amongst the researchers and a third party. The following search terms were employed to search PubMed, Medline and Scopus: ketamine abuse, ketamine abuse and surgery, ketamine and interstitial cystitis, ketamine abuse and interstitial cystitis, ketamine abuse and complications, ketamine and cystitis, ketamine abuse and cystitis, ketamine and cystoplasty, ketamine abuse and cystoplasty, ketamine abuse and ileocecocystoplasty, ketamine and ileocecocystoplasty, ketamine and enterocystoplasty, ketamine abuse and enterocystoplasty, ketamine abuse and augmentation enterocystoplasty, ketamine and augmentation enterocystoplasty, ketamine and augmentation cystoplasty, ketamine abuse and augmentation cystoplasty, ketamine abuse and bladder, ketamine and bladder, ketamine cystitis, ketamine‐induced uropathy and surgery, ketamine and urinary diversion, ketamine abuse and urinary diversion, ketamine and cystectomy, and ketamine abuse and cystectomy. In vitro, animal studies, letters to the editor and papers that did not evaluate surgical outcomes were excluded.

We extracted the following data from each article: type of article (e.g., case report, case series, retrospective observational studies and literature review), time period over which study was conducted, number of patients (male and female), age, symptoms, symptom scores, patient reported outcomes, duration of ketamine abuse, indications for surgery, type of surgery, surgical technique, alternative treatments, cystoscopic findings, histological findings, pre and post‐operative hydronephrosis, serum creatinine, maximum voided volume, maximum uroflow rate, post‐void residual, bladder compliance, urinary frequency, nocturia, cystometric bladder capacity, urinary tract infection, vesicoureteral reflux, maximum bladder capacity, pain, strictures, length of follow‐up, major complications, and reoperations. Major complications were defined as those requiring reoperation or death.

Early in the data collection phase, it became evident that the few cases that did exits in the literature often did not present enough data points from which physicians and/or researchers could draw conclusions. To address this, the quality of each paper was assessed and graded based on a modified point system established by Hartmann et al.^11^ This point system was not used to depict internal or external bias nor is it intended to criticize any of the included authors; it is simply a tool used to portray the dearth of published information about this disease complex and highlight areas of future study. The parameters we sought for efficacy in each paper were number of subjects, length of follow‐up, reported loss to follow‐up, selection criteria, baseline demographic/clinically relevant characteristics, description of intervention, description of efficacy outcome measures, subjective measures at follow‐up, semi‐objective measures at follow‐up, objective measures at follow‐up and efficacy defined with valid measures. For safety, we searched for reported general complications, methods for evaluating complications, prompted complications and description of loss to follow‐up. For each category mentioned by a paper, they received one point. The point system ranges from 0 (*worst quality*) to 15 (*best quality*). By consensus, the authors agreed that a minimum threshold of eight categories was needed for a paper to be potentially scientifically valid enough. We bolded the categories that we deemed necessary to meet a threshold score in Table 4. The more categories a paper mentioned, the higher the score and the more scientific validity.

To aggregate the data, weighted averages were taken of all continuous variables, represented as mean ± standard deviation; categorical data are represented by number and percentage out of the whole (%). Based on the data present in the literature, we found it impossible to distinguish the outcomes of the various reconstructive surgeries as several papers reported on patient populations that had received different surgeries without linking outcomes to those procedures. Furthermore, demographic information on nonsurgical patients included in surgical publications were included as a means of assisting in the physicians in the decision‐making process.

## 3 RESULTS

**FIGURE 1:**
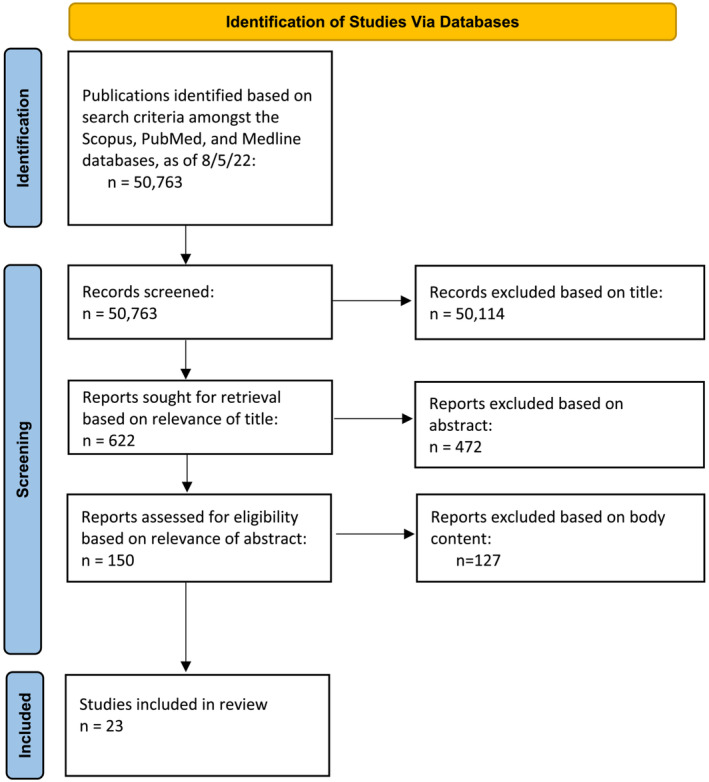
PRISMA flow diagram.

The search criteria yielded a total of 49 825 for Scopus, 748 unique articles in PubMed and 190 unique articles in Medline. Of the 50 763 identified articles, 622 were relevant based on title, 150 based on abstract, but only 23 papers were relevant by content (Figure 1). In all, 875 patients were documented as having ketamine cystitis, of whom 193 (22%) underwent reconstructive surgery (article descriptions can be found in Data S1). Demographic and the type of reconstructive surgery are depicted in Tables 1 and 2, respectively.

**TABLE 1:** Patient demographics.

Ketamine‐induced uropathy patients (*N* = 875)	Surgical (*N* = 193) (22%)	Nonsurgical (*N* = 682) (78%)
Males (*N* = 198)	84 (44%)	114 (17%)
Females (*N* = 92)	66 (34%)	26 (4%)
Sex unknown	109 (56%)	542 (79%)
Mean age (*N* = 330)	27.1 ± 2.2 (*N* = 150)	22.7 ± 1.9 (*N* = 180)
Duration of ketamine abuse in years (*N* = 323)	4.1 ± 1.3 (*N* = 173)	3.4 ± 0.6 (*N* = 150)

*Note*: *N* = the number of patients for whom data were available.

**TABLE 2:** Primary surgeries.

Primary surgeries (*n* = 193)	
Enterocystoplasty (EC)^a^	124 (64%)
Neobladder	10 (5%)
Urinary diversion	6 (3%)
Ureteroneocystostomy	15 (8%)
Continent urinary stoma	8 (4%)
Bladder autoaugmentation by transurethral vesicomyotomy (BATV)	12 (6%)

*Note*: *n* = the number of patients for whom data were available.

^a^ 112 simple EC; 10 simple cystectomies; 1 cystoprostatectomy; and 1 radical cystectomy. Numbers add up to >193 because some patients had combined procedures.

Overall, the quality of the studies reporting surgical outcomes was poor. Surgical success rates were available in only 110/193 patients (57%), and less than 50% of the patients had any outcome data with respect to pain, voids per day, bladder capacity and compliance or post‐void residual urine (Table 4).

Surgical outcomes are summarized in Table 3. Mean follow‐up was 20 ± 9.0 months, and ranged from 10 days to 35 months, but it was available in only 67% (130/193) of patients. Overall, the initial surgical success rate was 78% (88/110 patients), and the reoperation rate was 17% (19/110 patients), but it was not possible from the data to determine how many reoperations were done because of surgical failure versus complications. Major post‐operative complications occurred in 19/34 (56%) and 3/28 (11%) patients that underwent RLUTS were found to develop de novo ureteral obstruction. 19/78 (24%) required a reoperation to relieve pain and 19/73 (26%) required intermittent catheterization.

**TABLE 3:** Surgical results.

Surgical results	*n*	Pre‐op	Post‐op
Post‐op follow‐up (months)	130		20 ± 9.0
Patient reported success	110		88 (78%)
Pain	69	53 (77%)	16^a^ (23%)
Voids per day	68	28 ± 1.4	18 ± 2.8
Maximum voided volume (ml)	108	68 ± 24	265 ± 19
Bladder capacity (ml)	74	96 ± 61	330 ± 30
Bladder compliance	68	12 ± 2.5	49 ± 9.3
Post‐void residual urine (ml)	68	11 ± 3.6	61 ± 20
Hydronephrosis/ureteral obstruction	86	44 (51%)	7 (8%)
Vesicoureteral reflux	68	25 (37%)	5 (7%)

*Note*: *N* = the number of patients for whom data were available.

^a^ All patients with persistent pain reported relapse of ketamine abuse.

Table 4 quantifies the quality metrics for the identified articles. Only 60% of papers achieved our minimum validation threshold, and 53% commented on all categories we defined for safety and efficacy. This indicates that the overall quality of the studies was subpar at best. The grading criteria for data points contained in the papers regarding surgical outcomes in KU patients on average only presented 41% of the relevant data points necessary.

**TABLE 4:** Quality and safety outcome measures.

Category		Prevalence (%)	Article	Grade (%)
Number of subjects		100	Chen, et al.^15^	37%
Length of follow‐up		65	Cheng, et al.^22^	24
Reported loss to follow‐up		22	Chiew, et al.^23^	19
Selection criteria		100	Chung, et al.^18^	82
Baseline characteristics provided		100	Cottrell, et al.^7^	24
Description of intervention		96	Hanno, et al.^24^	20
Description of efficacy outcome measures		70	Hopcroft, et al.^25^	23
Used objective measures at follow‐up		83	Jhang, et al.^16^	74
Histological findings	Pre‐op	48	Jhang, et al.^26^	30
	Post‐op	30	Kidger, et al.^27^	22
Cystoscopic findings		67	Lee, et al.^19^	74
Serum Creatinine	Pre‐op	17	Misra, et al.^28^	34
	Post‐op	9	Mui, et al.^29^	26
MVV	Pre‐op	33	Ng, et al.^17^	44
	Post‐op	22	Peng, et al.^30^	59
Q Max	Pre‐op	15	Raison, et al.^31^	27
	Post‐op	20	Shahzad, et al.^32^	38
PVR	Pre‐op	17	Sihra, et al.^33^	44
	Post‐op	17%	Tan, et al.^20^	50
Compliance	Pre‐op	22	Wu, et al.^34^	49
	Post‐op	17	Yee, et al.^35^	37
CBC	Pre‐op	26	Yong, et al.^36^	29%
	Post‐op	20	Zhong, et al.^37^	33
ABC	Pre‐op	0	**Average**	39
	Post‐op	0		
MBC	Pre‐op	39		
	Post‐Op	35		
Use of semi‐objective measures at follow‐up		17		
Frequency	Pre‐op	39		
	Post‐op	28		
Nocturia	Pre‐op	15		
	Post‐op	17		
Used subjective measures at follow‐up		39		
Pain	Pre‐op	43		
	Post‐op	35		
Efficacy defined with valid measures		70		
General complications		30		
Methods for evaluating complications		0		
Prompted complications (questionnaire, anamnesis)		0		
Description of loss to follow‐up		10		
UTI	Pre‐op	17		
	Post‐op	26		
VUR	Pre‐op	22		
	Post‐op	24		
Ureteral obstruction	Pre‐op	4		
	Post‐op	15		
Reoperations		74		
Average of surgical outcome data points		41		
Threshold categories^a^		60		
Overall categories^a^		53		

Abbreviations: ABC, anaesthetic bladder capacity; CBC, cytometric bladder capacity; MBC, maximum bladder capacity; MVV, maximum voided volume; PVR, post‐void residual volume; Q Max, maximum flow; UTI, urinary tract infection; VUR, vesicoureteral reflux.

^a^ Category refers to data deemed necessary to assess the scientific validity of each paper.

## 4 DISCUSSION

Evident in our data is the fact that there is a dearth of information regarding its prevalence and health consequences that KU has on the lower urinary tract. Our search found only 23 out of 143 studies that mentioned this terrible disease complex, and the overall quality of even these studies was poor (Table 4).

Ketamine‐induced uropathy is characterized by severe LUTS that mimic end‐stage interstitial cystitis. The patients included in our study were found to complain of marked urinary frequency (as often as 20 to 30 voids per day), dysuria and bladder pain that intensifies with increasing bladder volume; the symptoms seemed to have been refractory to empiric treatments in end‐stage KU patients. Without exception, all of the data indicate that reconstructive surgery is effective for treating end‐stage KU, at least in the short term. After successful surgery, we have found that there to be resolution of bladder pain, hydronephrosis, ureteral obstruction, vesicoureteral reflux and a decrease in urinary frequency. Maximum voided volume, bladder capacity, bladder compliance and post‐void residual volume all improve (Table 3).

Cessation of ketamine is always the first and key step in the management of KU; Winstock et al.^12^ reported that KU symptoms resolved after cessation of ketamine abuse in 51% of patients. A study by Mak et al. showed that abstinence from ketamine by chronic users for 1 year has the potential to improve urinary symptoms (urgency and frequency) and increase voided volumes.^13^ Although no specific ketamine withdrawal syndrome has been reported in the literature as of yet,^14^ a recent study found that abstinence from ketamine may cause some nonspecific symptoms such as anxiety, poor appetite, drowsiness and fatigue.^15^

Medical treatment may be helpful in stopping disease progression and relieving symptoms in the early stages of KU, as seen in our nonsurgical patient population. Such therapies include antimuscarinics and nonsteroid anti‐inflammatory drugs, cystoscopic hydrodistension and intravesical instillation therapy with hyaluronic acid or heparin. Intravesical treatment with surface protectants has been shown to reduce bladder pain; however, it is difficult to effectively treat the symptoms if patients continue using ketamine.^16^ If surgical management is chosen, compliance with post‐operative care and abstinence should be stressed to the patient before surgery.^17^ In fact, all patients in this review who continued to experience pain post‐operatively, or required a reoperation, had continued to abuse ketamine during or after treatment.

Surgical reconstruction is indicated for those with end‐stage bladder disease characterized by refractory overactive bladder symptoms, bladder pain, low bladder capacity, and low bladder compliance. Of course, it is best accomplished before the onset of hydronephrosis and vesicoureteral reflux, but over half the patients included in this review already had hydronephrosis or vesicoureteral reflux at the time of their reconstruction.

These data are disconcerting because of the apparent rapid progression from the beginning of ketamine abuse to end‐stage bladder; there was only 1‐year difference between the duration of ketamine abuse in those patients who underwent surgery (4.4 years) and those who did not (3.4 years). This suggests that the time interval from the beginning of ketamine‐induced uropathy until end‐stage bladder and ureteral involvement may be measured in months or a year, confounding the decision making about the optimal time to consider lower urinary tract reconstruction. This is especially relevant in so far as low bladder capacity, low compliance, vesicoureteral reflux and hydronephrosis are not likely to be resolved after cessation of ketamine abuse.

Surgical options for those with end‐stage bladder include augmentation enterocystoplasty, partial or supratrigonal cystectomy with enterocystoplasty, bladder autoaugmentation by transurethral vesicomyotomy, bladder hydrodistention, simple cystectomy or cystoprostatectomy and creation of a neobladder or urinary diversion (conduit vs. continent diversion). The aims of these procedures are to alleviate symptoms and protect the kidneys from the hostile environment of the end‐stage bladder. What seems clear from these limited data found in our search is that all of the current reconstructive procedures appear to improve LUTS, relieve bladder pain (provided that the patient abstains from ketamine) and protect the kidneys,^18^ at least in the short term, unless ureteral obstruction occurs as a complication of the surgery itself. In the combined series presented herein, 64% of the patients underwent enterocystoplasty and the remainder underwent either cystectomy and neobladder, supravesical urinary diversion, bladder hydrodistention or bladder augmentation with transurethral vesicomyotomy.

Chung et al.^18^ and Lee et al.^19^ have reported on 14 and 26 patients, respectively, all of whom underwent augmentation enterocystoplasty (AE) to manage KU, with no significant peri‐ or post‐operative complications. The patients studied by Lee et. al.^19^ abused ketamine for an average of 5 years and developed severe lower urinary tract complications such as contracted bladder, hydronephrosis and vesicoureteral reflux. Cystometric bladder capacity, Qmax, voided volume and bladder compliance all significantly improved after AE. Hydronephrosis resolved in more than 75% of patients and vesicoureteral reflux resolved in all who underwent AE with ureteral reimplantation.^15^ Most importantly, all patients who stopped using ketamine were free of bladder pain post‐operatively. This study highlighted the effectiveness of AE in treating KU‐induced bladder pain and restoring normal lower urinary tract function.^19^

Recently, other procedures have been explored as management options for ketamine cystitis. In a particularly well‐designed study, Tan et. Al^20^ compared the efficacy of bladder autoaugmentation by transurethral vesicomyotomy (BATV) to bladder hydrodistention (BH). They found that BATV is superior to BH in terms of increasing urodynamic outcomes, specifically with respect to MCC, bladder compliance, Pdet max, and PVR. Both procedures have similar rates of complications as well as symptom relief established by validated questionnaires.^16^ The strengths of this study lie in its use of objective and subjective measures to report surgical outcomes. Its retrospective study design, small sample size and limited length of follow‐up are endemic to ‘orphan’ diseases but provide useful data about a novel solution to a most difficult problem. This study appears to offer two safe and possibly effective options for patients that avoid major urinary tract reconstruction; but as they say ‘more studies are needed’, to corroborate these results.

Based on the results of this review, we believe that it is safe to say, that after reconstructive surgery, bladder pain, urinary frequency and urgency, maximum voided volume, bladder capacity and compliance and maximum detrusor pressure all improve.^18^, ^21^ In addition, vesicoureteral reflux and hydronephrosis resolve. Of course, longer term follow‐up and higher quality studies are need to corroborate these results, but it is clear that after successful surgery, resumption of KA appears to uniformly result in refractory KU once again.

There were significant limitations to this review because of the overall poor quality of the studies themselves, the most important of which were that a quantitative estimate of post‐operative symptoms was available in less than a third of studies (mean = 22%, range = 0%–43%) And only 40% of studies even mentioned the length of follow‐up, with a range of 10 days to 35 months. In addition, many of the studies were not explicit about which surgeries were done in patients requiring reoperations or even which patients needed reoperations.

## 5 CONCLUSION

Ketamine‐induced uropathy is a rapidly progressive, devastating condition that predominantly affects teenagers and young adults. It can lead to end‐stage bladder within a few years after its onset. Once refractory LUTS set in, the only effective treatment is major reconstructive surgery ranging from augmentation enterocystoplasty to cystoprostatectomy and urinary diversion.

## AUTHOR CONTRIBUTIONS

All those that had contributed to the creation of this manuscript have been included in the author section.

## CONFLICT OF INTEREST STATEMENT

There are no conflicts of interest amongst any of the researchers.

## Supporting information


**Data S1.** Supporting Information
